# The Contributions of Empirical Evidence to Socio-Ethical Debates on Fresh Embryo Donation for Human Embryonic Stem Cell Research

**DOI:** 10.1111/j.1467-8519.2009.01792.x

**Published:** 2011-07

**Authors:** Erica Haimes, Ken Taylor

**Keywords:** embryos, hESC research, embryo donation, empirical bioethics, spare embryos, embryo donors

## Abstract

This article is a response to McLeod and Baylis (2007) who speculate on the dangers of requesting fresh ‘spare’ embryos from IVF patients for human embryonic stem cell (hESC) research, particularly when those embryos are good enough to be transferred back to the woman. They argue that these embryos should be frozen instead. We explore what is meant by ‘spare’ embryos. We then provide empirical evidence, from a study of embryo donation and of embryo donors' views, to substantiate some of their speculations about the problems associated with requesting fresh embryos. However, we also question whether such problems are resolved by embryo freezing, since further empirical evidence suggests that this raises other social and ethical problems for patients. There is little evidence that the request for embryos for research, in itself, causes patients distress. We suggest, however, that no requests for fresh embryos should be made in the first cycle of IVF treatment. Deferring the request to a later cycle ensures that potential donors are better informed (by experience and reflection) about the possible destinations of their embryos and about the definition of ‘spare embryos’. Both this article, and that by McLeod and Baylis, emphasize the need to consider the views and experiences of embryo donors when evaluating the ethics of embryo donation for hESC research.

## I. INTRODUCTION

This article is written in response to McLeod and Baylis^1^ who argue that ‘spare’ fresh embryos that are good enough to be transferred to the woman for treatment should not be donated^2^ to human embryonic stem cell (hESC) research, since doing so is not in the woman's self- or other-regarding interests. They conclude that this practice ‘is so fraught with difficulties that it simply ought not to take place’.^3^ Such embryos, they assert, should be frozen so that decisions about what happens to them can be taken at a time and place separate from the IVF process.

McLeod and Baylis raise some important issues: the need to consider the experiences of those being asked to donate embryos; the need to question what types of embryos are being donated (fresh or frozen; suitable for transfer or unsuitable for transfer); and whose interests those donations serve. Their analysis, however, is based on speculations about what *might* happen when the request to donate is made and how potential donors *might* reason their way through such requests. It is useful to compare these speculations with empirical evidence and evaluate what this evidence contributes to understandings of the issues raised.^4^ This is what we do in this article.

First we examine the term ‘spare’ when used with reference to embryos. Then we present empirical evidence from a UK Wellcome Trust-funded study on potential donors' experiences of being asked to donate embryos for hESC research. We then review the evidence on issues that arise following embryo freezing, to evaluate whether freezing is a solution to the concerns raised by McLeod and Baylis. There are many other points raised in their rich article that deserve attention but space constraints require this more specific focus. We reach similar conclusions about the need for patients to have ‘time and distance from their IVF treatment that would allow them to reflect carefully on whether they want to donate excess embryos’^5^ though we suggest different ways to achieve that opportunity for reflection.

## II. DEFINING ‘SPARE’

The term ‘spare’, with reference to embryos being donated for research, is a highly contestable concept. First, ‘spare’ can refer to the quality of the embryo (not good enough either for transfer or freezing). Second, embryos can be designated ‘spare’ because of constraints on options within any particular cycle within any particular clinic (such as when the woman has had the maximum allowable number of embryos transferred, and or when the clinic follows a freezing policy that sets limits on either the maximum or minimum number of embryos that can be frozen) even though the patient still wants to use them and might not consider them ‘spare’. Third, embryos can be designated ‘spare’ by patients who have finished their reproductive projects (because of success or deciding to end treatment). In addition, ‘spare’ might not mean ‘spare for research’: other infertile patients might prefer these embryos be donated for their treatment. Thus, the quality of ‘spare-ness’ is not inherent to the embryo but rather is dependent on the context in which this is considered a relevant designation and on the authority, power or interests of particular groups or individuals to apply that label in pursuit of certain goals.^6^

The designation of ‘spare’ is perhaps most contentious in the ‘constraints-on-options’ scenario described above as it has the most potential to conflict with the couple's own views and interests. It is this that McLeod and Baylis refer to when they discuss the donation of embryos that are good enough to be transferred or frozen for later use. Their contribution is important as few previous articles have addressed the issues around the possibility that embryos that are good enough either to be transferred or frozen for future use are being donated for research. Also, few bioethics articles have addressed the question of the relative scientific usefulness of these different types of embryos (fresh or frozen; good enough or not good enough for transfer) for hESC research. However, their proposed solution, of freezing these embryos, has its own difficulties.

## III. AN EMPIRICAL STUDY OF EMBRYO DONATION

This study was conducted in one fertility clinic in the UK. All couples undergoing IVF treatment over a 10 month period in 2005–2006 who were asked to donate ‘spare’ embryos for hESC research were contacted on behalf of the social researchers, approximately six weeks after receiving their pregnancy result, to request participation in an interview to explore their views and experiences. Forty-four in-depth interviews were conducted; most couples had consented to donate embryos at some point though not all consented to the full range of studies and not all donated in every cycle.

Choosing to donate, or not, is a decision positioned in relation to other aspects of interviewees' lives, including, most crucially, the decision to seek IVF treatment in the first place. Couples can only be asked to donate because they are going through IVF; for them, having ‘our baby’ is the primary concern and forms the backdrop to the request to donate embryos. They are, from their point of view, ‘IVF patients’ rather than ‘potential embryo donors’.^7^

Our study indicates some support for McLeod and Baylis' argument that providing for research embryos that are good enough to be transferred to the woman is against the woman's self-interest. This is reflected in what interviewees said once they had time to reflect on the IVF process and realize that some of their embryos might have been used for research. We use the phrase the ‘troubling third embryo’ to refer to these embryos, the discussion of which aroused puzzlement as to what had actually happened to them and triggered the most overt displays of distress.^8^ At least thirteen couples questioned what had happened to their top quality embryos that were neither transferred nor frozen. Some simply did not know what had happened:

I did think about it at the time and then it went out of [my] head but I will mention it now [short pause] … Three fertilized … ethically they could only put two back in, so obviously I had one still good egg that had fertilized … nobody asked me what I wanted doing with that. And that happened both times. I just [assumed], wrongly or rightly, that it wasn't worth their while freezing it ‘cos there was only one, so therefore rather than waste it, they would use it for research. But it was never actually mentioned. (IVF16:1088–1128).

This aspect of her treatment clearly continues to puzzle her. Another said: ‘… all three were at the same stage so I thought it was a shame to take two and leave one out but that's just the way it is. They can't put back any more than that because it would be unsafe, so what can you do?’ (IVF3:869–885). Her tone of resignation and regret is echoed by another couple:

The three that were good to use, they had said they chose what they thought were the best two. So, at the time, no, you don't consider about the third one. Now we can [perhaps] think, ‘if that one had been put in [perhaps] that one would have worked’ whereas the other two hadn't. But there wouldn't be any point in doing that … (IVF19:1018–1064)

Another said about the third embryo, ‘you can't help thinking at the back of your mind, ‘that might be my one chance of having a baby and I've given it away for this research’ (IVF6.2: 351–396).

Others were less accepting. One woman said she had produced ‘six really good quality [embryos] and we had two go back … well, I signed the form, I signed the form, that was [my] decision to make [short pause]. But it's the good ones that upset me, the four little good ones’ (IVF18: 1718–1741). Earlier she had reflected that on both cycles of treatment, some embryos had deteriorated overnight and had not been good enough to freeze and therefore, ‘The second [cycle], I just wished I hadn't signed the form, just for a comparison, to see if there would have been just that one more, enough to be able to freeze’ (IVF18:437–446). This woman is taking responsibility for her decision to sign the consent form to donate embryos to research whilst also raising questions about the consequences of having done so. Her continued uncertainty about these consequences, after several cycles of treatment, suggests either a problem with the consent procedures or in her understanding of the decision-making around donation.^9^

Uncertainty featured in other couples' experiences:

I know they'll only put two back but when they say to you, ‘there's three good quality embryos suitable to put back in, so we've picked two of them’, you think, ‘there's only one, that one left might have worked’ … I know the people that are actually doing the procedure know best but they should still give you the option whether or not you want to put the three back in if you've only got three … if there's only three and they're saying that they're good enough to go in, but you can only put two in and the other one, ‘sorry we can't freeze one’ then they should give you the option of putting it in. (IVF13:1411–1497)

Another woman described how her realization that good embryos could go to research emerged, as she went through treatment and then reflected afterwards. We quote her at length to illustrate the development of her thinking over time. Referring to the early stages of the IVF process she said:

… at that stage it all seemed fairly straightforward to me. The only time I found it much harder was when there was a fertilized egg that they couldn't freeze, and I wasn't happy with it at that stage … that possibility had not occurred to me – that you'd have a viable embryo that they would not freeze … (IVF14:130–147).

When asked about how she compared the transferred to the non-transferred embryos she said:

At that point? I was worried about the actual [transferred] embryos at that point and it was only afterwards I thought, ‘that [non-transferred] embryo would be going for research’ and that if I'd thought about that at the time I probably wouldn't have been so happy … if they'd been rotten embryos, and not going to do anything, then that would have been something [else] but they were saying that that one was okay … I just thought that was something I had to come to terms with … I found it silly that I should find that harder when … there was no way I could get that [embryo] saved … There was nothing I could do for it. I wasn't really quite as clinical or sensible as I thought I was … I'd never thought of that possibility … I didn't know how I was going to feel about anything to be honest, and it was all very, very new … I was prepared for every other step of not getting enough eggs, or not getting any fertilized eggs, but I wasn't actually prepared for that step of having to throw one away. (IVF14: 155–243)

Later she made her point more strongly:

I felt differently about donating the viable embryo … because the way I felt it had been worded was, or how I understood it, was all the viable ones would be frozen and I'd obviously not understood that that might not happen. The non-viable embryos that weren't suitable for freezing and the eggs that didn't fertilize, I had no problems with, but the viable embryo, yes I did … I don't think I had really appreciated the emotional aspect of [long pause] the emotional aspect of, the wasting my own eggs, if you see what I mean? That *that* was a loss … (IVF14: 469–591)

This woman was pregnant at the time of interview so her words counter the view that pregnancy eliminates any difficulties experienced with IVF in general or in being asked to donate to research. These elements of the treatment and consent process were raised at an unusually early stage in this interview, compared to others, indicating that the issue was still very pertinent for her. The language of ‘saving’ the embryo and of ‘loss’, alongside the references to ‘feeling silly’ and not being so ‘clinical’ suggest a depth of feeling for which she had not been prepared. She makes an honest distinction about the consent process, between both how it was worded and how she had understood it, through which she takes responsibility for her decisions.^10^ Clearly it is preferable if consent procedures do not leave room for these sorts of misunderstandings or uncertainties.

Other data indicate that, perhaps not surprisingly, some couples do think about all the non-transferred embryos that *might* have worked, but the above data show that those with more than two good quality embryos face a starker contrast between embryos that had had quite a good chance of working (at least as good as the two that were transferred) and those that had never had much chance, being of poorer quality. It is then an additional burden to realize that the third (fourth/fifth) good quality embryo(s) might have gone to research instead, especially when other data suggest that the nature of that research is something they would rather not contemplate.^11^

Several couples questioned whether some embryos were *deliberately* not transferred or frozen *in order that* they could go for research. One couple speculated apologetically:

… it's now the third time … your mind does start racing and you start thinking about what's happened and how things have worked. [short pause] It's upsetting really to think that possibly on that first occasion we did have these seven embryos and they weren't frozen. Now from my memory they were all decent embryos … I seem to remember getting told that we had nine decent ones. (IVF28: 452–481)

Another couple was very direct:

… four times we've been through the cycle we've never had any frozen. And that was one of our concerns – that they weren't being frozen because they were wanting them for the research … they never actually [came] back and said they weren't good enough to freeze but they were good enough for research, or they weren't good enough for research either … they just said the quality was no good and that was all. Nothing more was said. The quality isn't good enough to freeze them, they wouldn't survive the process of freezing. (IVF15:387–420)

Whilst this might not be a case of good quality embryos going to research it is worth noting that the dual processes of treatment and research raised doubt in some patients' minds.

Others wondered if embryos had not been frozen as a direct consequence of having consented to donate. One couple had decided to donate only embryos that were not of good enough quality to transfer but then had doubts as to what actually happened:

There were discrepancies in my mind as to what was the right quality and what wasn't. And I felt like the second time I didn't want to donate them. … I don't know whether I was just all emotional or hormonal … but I felt like there'd been a bit of a conspiracy to get our [embryos] because we'd signed this form. And I know there probably wasn't but at the time … you don't know where you're at, it's such an emotionally charged time … I felt like things happened that shouldn't have happened and I felt like we didn't get to know exactly what went wrong. (IVF18: 326–385)

That they were puzzling over this raises questions as to whether these interviewees trusted the clinical team to prioritize their interests.^12^

These data endorse McLeod and Baylis' concerns about the donation to research of fresh embryos that would have been good enough to use in treatment. In summary, interviewees' concerns encompass: doubts about whether the best embryos were transferred; confusion about what happened to the good-enough-to-be-transferred embryos that were not transferred; feelings of regret about the waste of good embryos; whether they should have insisted that these embryos be frozen; an emergence of suspicion that the clinic might be prioritizing research over treatment, and whether consenting to donate to research meant that they were not being given the best possible chance of getting pregnant. It is also clear that they only realized that they might have donated good embryos to research after the cycle had ended: they did not fully appreciate at the time of consenting to donate that this possibility might arise. They mention the challenges of the IVF process, particularly during the first cycle, and there being so much going on that it was difficult to understand all that was happening.

Clearly the timing and nature of the consent process play a crucial part in shaping potential donors' understandings and responses. It is for these, and other reasons (see below) that McLeod and Baylis argue that IVF patients should not be asked to donate fresh embryos. Rather, they argue, patients should be given the opportunity to freeze any surplus embryos and then decide whether they want to donate these after careful reflection: ‘patients are better off freezing their excess fresh embryos, rather than donating them to research’.^13^

From the above data we have some sympathy for this view and for the view that freezing provides time for patients to reflect and develop an understanding of what it is they are being asked to do. However freezing then thawing embryos creates other problems, which McLeod and Baylis do not consider. The fact that embryo freezing is a popular option^14^ does not make it an efficacious one. In order to evaluate the different ways of protecting donors' interests the empirical evidence on the costs and benefits for patients of freezing needs to be reviewed.

## IV. THE COSTS AND BENEFITS OF FREEZING EMBRYOS

In addition to McLeod and Baylis' arguments, the rationale for freezing good embryos is that this reduces the need for women to undergo ovarian stimulation and egg retrieval in later cycles. This reduces the health risks of hyperstimulation, reduces the psychological stress of the IVF process on women, and improves the cumulative pregnancy rate.^15^ It also saves time and money, for clinics and patients.

However, there are many questions to ask about freezing embryos: is it safe; how effective is it in producing successful pregnancies; could freezing raise false hopes; could freezing even lead to women undergoing more cycles of treatment; and is it financially worthwhile? Also, does freezing have other emotional, ethical or social costs and, if so, who bears them?

Although embryo-freezing is a well established practice, there is surprisingly little published material addressing these questions. Also, in the UK it is difficult to obtain reliable information about the freezing policies different clinics adopt and on their management of frozen embryos. Authors comment on the variability between individual clinics, between and within countries, and on the cultural variations whereby different countries allow different things to be done or not done with frozen embryos.^16^ Estimates suggest that, in 2002, 400,000 frozen embryos existed in the USA, 52,000 in the UK and over 71,000 in Australia.^17^ Baylis et al.^18^ report that in 2003 the 54% of Canadian fertility centres that responded to a request for data held 15,615 embryos. These numbers are significant and highlight the need to research the questions we raised above. Lyerly et al.^19^ also note that public debate lacks empirical data on the attitudes of those who have to make decisions about these embryos.

Bankowski et al.^20^ report that cryopreservation is generally safe, though there are hazards associated with viral contamination, clerical errors on labelling embryos, and accidental destruction of frozen embryos. This general level of safety means that most debates around cryopreservation focus on the ethics of embryo disposition rather than on freezing itself. These authors also note that in 2002, 17.9% of all transfers in the USA were performed using cryopreserved embryos and that embryo cryopreservation had increased the ongoing pregnancy rate per oocyte retrieval by 6.6% and lowered the cost per delivered baby to between 25%–45% of the cost of a fresh cycle.

These financial and other benefits should be seen in the context reported by Lyerly and Faden^21^ that in 2003 in the USA only 65% of embryos survived thawing. Reports from UK clinics^22^ suggest these figures have changed little over the last five years. Of 24 clinics providing information on freezing, only 18 reported survival rates, post-thawing; these ranged from 50%–80% with the majority reporting between 60%–70%. Ten clinics indicate that pregnancy rates using frozen- thawed embryos are significantly reduced compared with fresh embryo transfer cycles, though only three provide figures, quoting 10%–20% against pregnancy rates of between 30%–40% using fresh embryo transfer. Therefore an IVF couple might not have quite as many ‘banked’ embryos as hoped and there is the danger that freezing raises false hopes about the ability of frozen embryos to produce a pregnancy.^23^

Of course the reduction in viability can be set against the removal of the risks of ovarian hyperstimulation. While it is true that most of the risks of IVF are removed if the thawed embryos are replaced in a natural cycle, for many women replacement will take place during cycles mediated by the same range of drugs required during transfer of fresh embryos, albeit without hyperstimulation and egg retrieval. The reduction of viability following freezing could also lead women to require more cycles of treatment rather than fewer if, as is likely using thawed embryos, treatment is not successful and several cycles are undertaken using up the frozen embryos before embarking again on a full cycle of treatment. Thus there is still some risk and burden faced by women.

Practicalities, such as the costs of storing frozen embryos, need to be considered.^24^ De Lacey claims that the introduction of a fee for freezing ‘was associated with’ a threefold increase in patients deciding to destroy their frozen embryos.^25^ Most, though not all, clinics in the UK charge patients for freezing and storage of embryos, ranging in late 2008 from free to £800 for freezing and a further £200–£500 per annum for storage. One final, practical, consideration is whether freezing is legally available. Leach Scully and Rehmann-Sutter^26^ note that cryopreservation of IVF embryos, for any purpose, has been forbidden in Switzerland since 2001.

Equally important, in view of McLeod and Baylis' concerns, is whether embryo freezing has other emotional, ethical and social costs, and, if so, who bears these costs? There is growing evidence that this is the case. First, there is the problem of unclaimed or unused frozen embryos.^27^ Nisker et al. (2006) suggest that some couples who had previously consented to donate frozen embryos to research changed their minds when contacted specifically to request donation to hESC research.^28^ Newton et al.^29^ found that ‘a substantial number of patients will not return for stored embryos’ and noted that many patients will not provide a final directive for embryo disposition even when re-contacted. As Bankowski et al.^30^ report, patients can choose inaction, keeping embryos stored in preference to making any other decisions.

Second, de Lacey^31^ argues that freezing raises moral and legal problems, given that patients' circumstances change over time and different issues arise, such as who owns the embryos and who has the right to decide what should be done with them, and after how long a time period. Fuscaldo et al. say that most participants in their study of couples with embryos in storage ‘described the process of making a decision about surplus embryos as difficult and emotionally fraught’;^32^ their findings concur with those of Nachtigall et al., McMahon et al. and of de Lacey^33^ who says that once couples had decided that they would not use the embryos for themselves, the decision about what to do with them ‘was much harder than they imagined and fraught with moral ramifications.’ In a later article de Lacey argues that the ‘process of determining an outcome for frozen embryos is a complex human experience, the depth of which has not yet been fully described’.^34^

Whilst some argue from empirical evidence that these difficulties can be overcome,^35^ the stress of the decision making needs to be compared with the stress which McLeod and Baylis speculate arises from not having the option of freezing. Lasting effects of cryopreservation on psychosocial health need further study.^36^ Account also has to be taken of those embryos that are disputed over in cases of divorce, death, and laboratory or clinic error.^37^ Stress is added by the fact that advance directives about what couples want to happen to their surplus frozen embryos prove to be an unreliable indicator of what they finally decide.^38^ Preferences for disposition are based on changing information and experiences and are therefore ‘dynamic’.^39^

In one study some patients wondered if they chose the option of freezing out of an inappropriate level of trust in the clinical team and an ‘uncritical acceptance’ of cryopreservation.^40^ Many interviewees ‘found … none of the available choices for embryo disposition was ideal or even acceptable’.^41^ These authors conclude, ‘Clinicians should understand that IVF patients' willingness to participate in embryo cryopreservation does not necessarily indicate a reasoned and reflective decision to do so’.^42^ Patients should be warned of the possible ‘burden of facing what may be a morally difficult decision in the future’ and it should be acknowledged that they are likely to change their minds about disposition over time.^43^

De Lacey concludes that ‘choosing a destiny for frozen residual embryos is a challenging and complex process’.^44^ Clearly freezing buys time and that is not an insignificant consideration; there is, for example, a degree of reversibility in the decision to freeze (at least for the 60%–70% of embryos that thaw successfully) that is not present in the decisions to discard or to give to research or treatment of others.^45^ However, the problems arising from freezing that have been described hitherto have to be balanced against these benefits. Taken as a whole, these data and observations suggest that a cautious approach by fertility clinics towards freezing is a reasonable strategy.

## V. CONCLUDING COMMENTS

In agreeing with McLeod and Baylis about patients' needs for time, reflection and distance, but in having some reservations about freezing as a means to that end, we have the following suggestions to make.

Our first suggestion is that clinics requesting embryos for hESC research (or considering entering into such practices) could follow different practices for the first and subsequent cycles of IVF. We suggest that patients are not asked to donate fresh embryos for hESC research in their first cycle of IVF, since this is experienced by patients as a particularly stressful and confusing time. A delay in making research requests for fresh embryos would at least ensure that the stress of IVF treatment, coupled with the sense that there is so much going on in the first cycle^46^ and the variable meanings that IVF patients attach to embryos as they go through that process,^47^ would all be experienced before the need to consider other decisions about research. Therefore, when those requests do arise, the potential providers are likely to be more fully aware of the possible consequences for themselves. Deferring the request to a later cycle would also reduce the problem of the ‘troubling third embryo’, as patients would be better informed by experience and reflection, as well as by documentation, about the possible uses of their embryos, whatever the quality. Similarly, the definitions of ‘spare embryos’ would be clearer, since patients will be better equipped either to query this term or even to negotiate their preferred definition with the clinics. The deferring of requests until the second cycle would also reduce or remove the suspicion that ‘research comes first’ in any of those clinics providing embryos for stem cell scientists. Consequently the ethical protection of patients would be enhanced.

There are of course practical consequences of this suggestion. If only two (or one, under single embryo transfer protocols) embryos are transferred back to the woman there might be other good quality embryos remaining. The decision in the first cycle over what should happen to these should rest with the patients, given the time, physical and emotional effort, and money they have invested in their production. This could lead to a marginal increase in numbers frozen after first cycles which might be considered an odd outcome, given the concerns that have been raised in this paper so far about the problems associated with freezing. However, there are ways to avoid an increase in the overall total of embryos frozen across all cycles (see below) and anyway this marginal increase would not equal the numbers frozen if McLeod and Baylis' suggestions are followed. There would also be a reduction in the number of fresh embryos made available for research, though again the suggestion below might counter the effects of this.

Just as we suggest the first cycle follow a conservative policy on research and a liberal policy on freezing, we suggest that the second and subsequent cycles follow a liberal policy on research and a conservative policy on freezing. That is, that the request to provide embryos for research should be allowed, since there is little evidence that this in itself causes patients distress.^48^ The embryos requested might come from frozen stores or be fresh, from new treatment. Patients who feel better informed, feel that their needs are being protected, and are less suspicious about the priorities of clinics, might well be happier about donating fresh embryos in subsequent cycles. Patients' attitudes towards the supply of embryos (defined as ‘spare’ according to criteria agreed with patients) might well be more consistent across cycles in the future.

Whilst freezing would still be made available in second and subsequent cycles, the dis-benefits of freezing need to be more fully explored with patients than seems currently to be the case. It could be argued that a cautious policy on freezing should be more widely followed by all clinics in second and subsequent cycles, to avoid the build up of the many problems described earlier.^49^ If the practice of mild ovarian stimulation^50^ gains acceptance it might well be the case that fewer embryos are produced in the first place and the dilemma of freezing, or not, is reduced for both patients and clinics.^51^

Fertility treatment is a notoriously tricky area to negotiate, in both practical and ethical terms: its history is peppered with practices that were initially seen as solutions to problems but subsequently became the source of problems themselves. The practices of embryo freezing and requesting embryos for stem cell research are no different. Having based the above suggestions on empirical evidence, we also advocate, of course, that the ethical evaluation of any new patterns of practice along the lines suggested is also informed by empirical analysis. However, when navigating routes through and around the many issues that need to be considered when exploring the problems discussed in this article, the most important consideration, we suggest, should be the ethical protection of patients.

## Footnotes

1 McLeodCBaylisFDonating Fresh Versus Frozen Embryos to Stem Cell Research: in Whose Interests?Bioethics2007214654771792762310.1111/j.1467-8519.2007.00592.x

2 ‘Donation’ is an increasingly problematic term since it implies certain motivations: Haimes has argued for the use of ‘provision’ as a less value-laden term; E. Haimes. Acquiring and providing human organs, gametes and tissue in the clinical context: socio-cultural issues. *Presentation to the Nuffield Council on Bioethics Workshop: Volunteering, donation and payment in the clinical context* December, 2008, London.

3 McLeod & Baylis, *op. cit.* note 1, p. 477.

4 HaimesEWhat Can the Social Sciences Contribute to the Study of Ethics?Bioethics200216891131208315710.1111/1467-8519.00273HaimesEWilliamsR‘Sociology, Ethics and the Priority of the Particular: Learning from a Case Study of Genetic Deliberations’Br J Sociol2007584574761772750310.1111/j.1468-4446.2007.00160.xMolewijkBFrithLEmpirical Ethics: Who Is The Don Quixote?Bioethics200923iiiv1933851910.1111/j.1467-8519.2009.01707.x

5 McLeod & Baylis, *op. cit*. note 1, p. 477. See also JNiskerDevelopment and Investigation of a Free and Informed Choice Process for Embryo Donation to Stem Cell Research in CanadaJ Obstet Gynaecol Can20061090390810.1016/S1701-2163(16)32279-417140508

6 See also; HolmSThe Spare Embryo – a Red Herring in the Embryo Experimentation DebateHealth Care Anal1993163661013435910.1007/BF02196973SvendsenMKochLUnpacking the Spare Embryo: Facilitating Stem Cell Research in a Moral LandscapeSoc Stud Sci20083893110

7 HaimesETaylorKThe Experiences and Values of IVF Couples Who Were Asked to Donate Fresh Embryos for Human Embryonic Stem Cell Research: Their Views on EmbryosHum Reprod200924214221501950261610.1093/humrep/dep124PMC2727400

8 Reference to the ‘third’ embryo is shorthand: in the UK usually only two embryos can be transferred back to the woman in any single cycle of treatment; if she has more than two good quality embryos these can, in theory, be frozen. At the time of our research, this particular clinic had had a longstanding policy of freezing a minimum of four top quality embryos in any one cycle. This policy was rooted in a cautious view of the efficacy of using frozen-thawed embryos in treatment, so some couples could have had up to three top quality embryos that were neither transferred nor frozen. IVF is still an evolving field and elements of that policy have since changed.

9 We note however that there is some uncertainty in the details of her response anyway since if she had had four good quality embryos remaining they would have qualified, numerically, for being frozen.

10 The practicalities of fieldwork meant that we were unable to observe the consenting procedures so cannot comment directly on this but the willingness of many interviewees to take responsibility for their decisions to donate deserves further attention, especially in light of McLeod and Baylis' concern that women might be coerced or exploited.

11 Haimes and Taylor 2009, *op. cit.* note 7, p. 2147.

12 It should be noted, however, that the majority opinion about the clinic, the staff and the treatment received, though not blindly uncritical, was very positive.

13 McLeod & Baylis, *op. cit*. note 1, p. 469.

14 Ibid: 469.

15 LyerlyADFactors that Affect Infertility Patients' Decisions About Disposition of Frozen EmbryosFertil Steril200685162316301667817810.1016/j.fertnstert.2005.11.056BankowskiBThe Social Implications of Embryo CryopreservationFertil Steril2005848238321621382910.1016/j.fertnstert.2005.02.057

16 Gurmankin and Caplan, 2004, cited in de LaceySPatients' Attitudes to Their Embryos and Their DestinyBest Pract Res Clin Obstet and Gynaecol2007211011121704553010.1016/j.bpobgyn.2006.08.003 p. 109; Leach ScullyJRehmann-SutterCCreating Donors: the 2005 Swiss Law on Donation of Spare Embryos to hESC ResearchJ Bioeth Inq200638193

17 Lyerly et al., *op. cit*. note 15, p. 1623; Bankowski et al., *op. cit*. note 15, p. 823; de Lacey, *op. cit.* note 16, p. 103.

18 BaylisFCryopreserved human embryos in Canada and their availability for researchJ Obstet Gynaecol Can200325102610311466353710.1016/s1701-2163(16)30344-9

19 Lyerly et al., *op. cit*. note 15, p. 1623.

20 Bankowski et al., *op. cit.* note 15, pp. 824–825.

21 LyerlyADFadenRWillingness to Donate Frozen Embryos for Stem Cell ResearchSciencexpress200721 June210.1126/science.114506717588897

22 Using information obtained from links to clinics on http://www.hfea.gov.uk[Accessed 3 April 2009].

23 Lyerly et al., *op. cit.* note 15, p. 1626.

24 Lyerly et al., *op. cit.* note 15, pp. 1625–1626.

25 De Lacey, *op. cit.* note 16, p. 104.

26 Leach Scully & Rehmann-Sutter, *op. cit*. note 16.

27 Bankowski et al. *op. cit.* note 15; FuscaldoGRussellSGilliamLHow to Facilitate Decisions About Surplus EmbryosHum Reprod200722312931381802502810.1093/humrep/dem325KlockSSheninSKazerRThe Disposition of Unused Frozen EmbryosN Engl J Med200134569701143996210.1056/NEJM200107053450118

28 Nisker et al. *op. cit.* note 5.

29 NewtonCChanges in Patient Preferences in the Disposal of Cryopreserved EmbryosHum Reprod200722312431281796221210.1093/humrep/dem287 3127.

30 Bankowski et al., *op. cit.* note 15.

31 de Lacey, *op. cit.* note 16.

32 Fuscaldo et al. *op. cit.* note 27, p. 3137.

33 NachtigallRDParents' Conceptualization of Their Frozen Embryos Complicates the Disposition DecisionFertil Steril2005844314341608488610.1016/j.fertnstert.2005.01.134PMC2811165McMahonCAMothers Conceiving Through IVF: Siblings, Setbacks and Embryo Dilemmas After Five YearsReprod Tech200010131135de LaceySParent Identity and ‘Virtual’ Children: Why Patients Discard Rather Than Donate Unused EmbryosHum Reprod200520166116691576095710.1093/humrep/deh831: p. 1664.

34 de LaceySDecisions for the Fate of Frozen EmbryosHum Reprod200722175117581741691810.1093/humrep/dem056: p. 1752.

35 Fuscaldo et al. *op. cit.* note 27; Lyerly et al. *op. cit.* note 15.

36 Bankowski et al., *op. cit.* note 15.

37 de Lacey, *op. cit.* note 16.

38 Klock et al., *op. cit.* note 27; Newton et al., *op. cit.* note 29.

39 Bankowski et al., *op. cit.* note 15; Nachtigall et al., *op. cit.* note 33.

40 Lyerly et al., *op. cit.* note 15, p. 1626.

41 Ibid: 1627.

42 Ibid: 1629.

43 Ibid: 1629.

44 de Lacey, *op. cit.* note 16, p. 109.

45 We are grateful to one of the anonymous reviewers for raising this point.

46 CohenCBUse of ‘Excess’ Human Embryos for Stem Cell Research: Protecting Women's Rights and HealthWomen's Health Issues20001211261082854710.1016/s1049-3867(00)00043-8

47 HaimesEPorzRLeach ScullyJRehmann-SutterC“So, what is an embryo?” A comparative study of the views of those asked to donate embryos for hESC research in the UK and SwitzerlandNew Genet Soc20082711312610.1080/14636770802077041PMC341954622904673

48 Whilst the patients quoted in this paper express concern about the ‘troubling third embryo’ going to research and in so doing suggest that the consent procedures could have been clearer, neither they nor other interviewees raised questions about the appropriateness or otherwise of the clinic requesting embryos for research. More detailed data from this study can be found in Haimes and Taylor (2009) *op. cit.* note 7, p. 2147.

49 Alternatively the global practice of freezing could be more rigorously regulated in the hope of removing the dilemmas described in this paper. Even if this was thought to be a desirable goal (and there are arguments both for and against such a goal that word limits prevent us exploring here) the difficulty of accessing reliable information about freezing practices and policies, in the UK let alone elsewhere, suggests that this would not be easily attained. Baylis et al. *op. cit.* note 18.

50 CollinsJMild Stimulation for *In Vitro* FertilizationHum Reprod Update200915131909175310.1093/humupd/dmn054

51 Equally it might well be the case that more frozen embryos are made available for research since the later the stage at which the request for embryos for research is made, the greater the chance that at least some of the IVF patients will have been successful in getting pregnant, or will have thought differently about their frozen embryos.

